# Different exosomes are loaded in hydrogels for the application in the field of tissue repair

**DOI:** 10.3389/fbioe.2025.1545636

**Published:** 2025-03-03

**Authors:** Yanyan Zhang, Wenjing Yan, Le Wu, Zihao Yu, Ying Quan, Xin Xie

**Affiliations:** College of Life Sciences, Northwest University, Xi’an, China

**Keywords:** exosome, hydrogel, tissue repair, regenerative medicine, controlled release

## Abstract

Exosomes are double-membrane vesicular nanoparticles in the category of extracellular vesicles, ranging in size from 30 to 150 nm, and are released from cells through a specific multi-step exocytosis process. Exosomes have emerged as promising tools for tissue repair due to their ability to transfer bioactive molecules that promote cell proliferation, differentiation, and tissue regeneration. However, the therapeutic application of exosomes is hindered by their rapid clearance from the body and limited retention at the injury site. To overcome these challenges, hydrogels, known for their high biocompatibility and porous structure, have been explored as carriers for exosomes. Hydrogels can provide a controlled release mechanism, prolonging the retention time of exosomes at targeted tissues, thus enhancing their therapeutic efficacy. This review focuses on the combination of different exosomes with hydrogels in the context of tissue repair. We first introduce the sources and functions of exosomes, particularly those from mesenchymal stem cells, and their roles in regenerative medicine. We then examine various types of hydrogels, highlighting their ability to load and release exosomes. Several strategies for encapsulating exosomes in hydrogels are discussed, including the impact of hydrogel composition and structure on exosome delivery efficiency. Finally, we review the applications of exosomes-loaded hydrogels in the repair of different tissues, such as skin, bone, cartilage, and nerve, and explore the challenges and future directions in this field. The combination of exosomes with hydrogels offers significant promise for advancing tissue repair strategies and regenerative therapies.

## 1 Introduction

Tissue injury generally refers to structural and functional changes in biological tissues caused by physical, chemical, or biological factors. Injuries can be classified into two main types: acute injuries (such as trauma or burns) and chronic injuries (such as chronic inflammation or long-term ischemia). Tissue injury affects the lives of millions of patients, leading to long-term disability, chronic discomfort, and suffering, which significantly undermines their quality of life. Due to the limited capacity of the human body for tissue repair, tissue healing and regeneration have long been significant challenges in the medical field when treating injuries (Niu et al., 2019). Currently, the main treatment methods for tissue injury are medication and surgery. While these approaches provide some relief, their effectiveness is often limited. The efficacy of pharmacological treatments still needs improvement (Khan and Scott, 2009). And surgical treatment has technical limitations, with the potential for postoperative complications (Zhang et al., 2021; Gao et al., 2024). Therefore, exploring suitable methods for tissue repair and regeneration has garnered significant attention from researchers.

With the development of tissue engineering, cell therapy has brought new hope for faster and more effective tissue repair. Cell therapy involves utilizing stem cells or cell-derived cells, which are transplanted into the body using specialized techniques to replace or repair damaged cells, tissues, or organs. Mesenchymal stem cells (MSCs) are particularly effective in promoting cartilage regeneration due to their immune regulation, anti-inflammatory properties, and regenerative capabilities. Human embryonic stem cells (ESCs) and induced pluripotent stem cells (iPSCs) have broad potential in cardiac regeneration due to their ability to differentiate into cardiomyocytes and endothelial cells (Zeng et al., 2023). However, stem cell therapy also carries risks, including tumorigenicity and immune rejection, which can limit its application in tissue repair (Mancuso et al., 2019).

**FIGURE 1:**
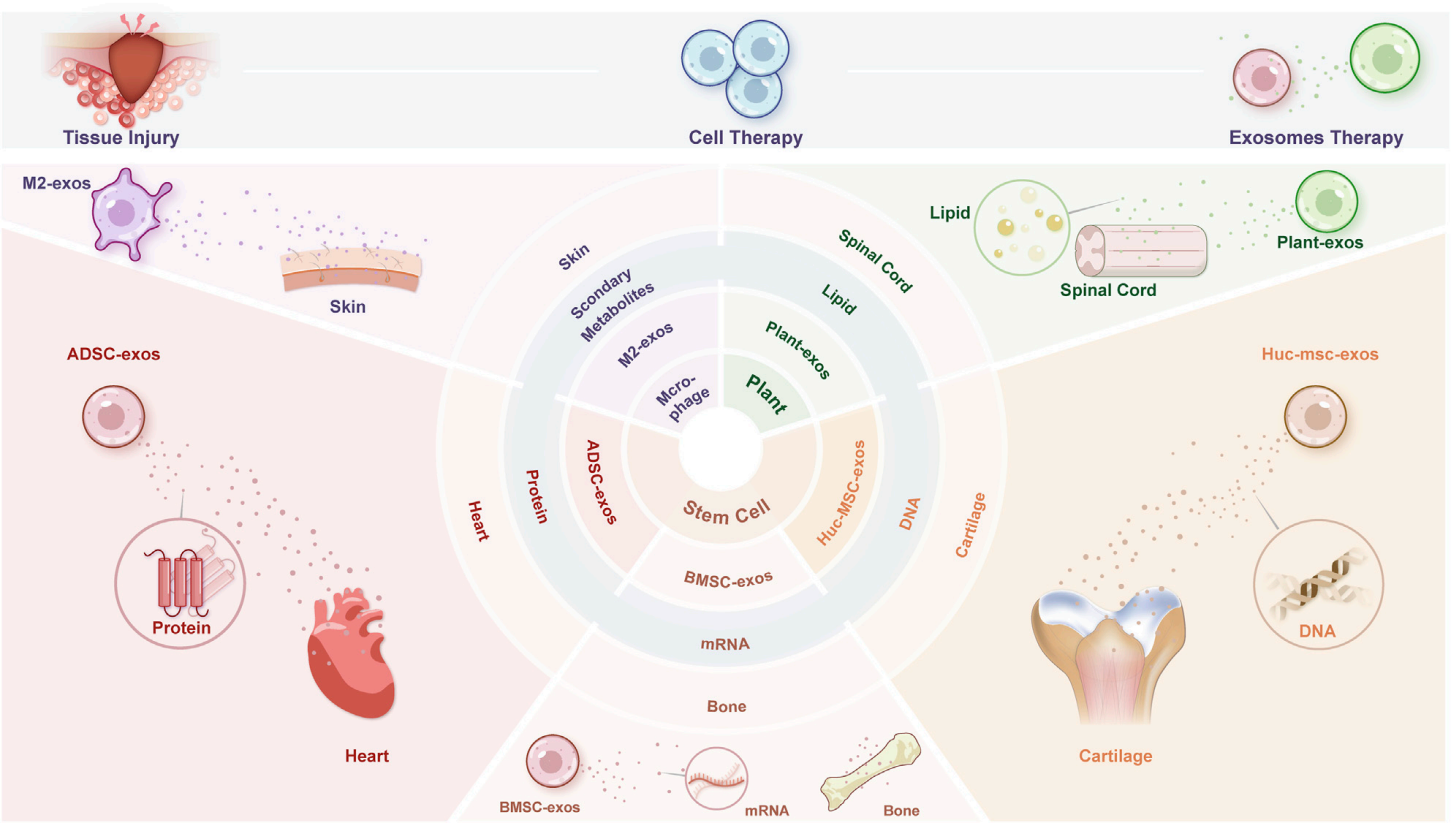
Graphical abstract of the review.

Cell-derived exosomes possess regenerative and repair capabilities similar to those of stem cells, but without the risks of tumorigenicity and immune rejection, enhancing safety and offering new hope for tissue repair and regeneration. Exosomes are small vesicles secreted by cells, typically ranging from 30 to 150 nanometers (nm) in diameter. They facilitate the transfer of signaling molecules, such as proteins, lipids, and RNA, and play a crucial role in intercellular communication, immune regulation, and tissue repair (Guan et al., 2022; Long et al., 2022). To better illustrate the functions of different exosomes in tissue repair, we have included aschematic diagram (Figure 1) that succinctly summarizes the multiple roles of exosomes. Although exosomes have the ability to promote tissue repair, free exosomes are cleared too rapidly from the body, while tissue repair often requires a complex and prolonged process. Therefore, the sustained action of exosomes is essential to meet the conditions necessary for effective tissue repair (Monguió-Tortajada et al., 2021) (Yang et al., 2021; Fan et al., 2022).

As a type of tissue repair scaffold, hydrogels can achieve sustained and targeted delivery of exosomes by loading them. Hydrogels are highly hydrophilic three-dimensional (3D) network structures that swell rapidly in water while retaining a large volume of water without dissolving. They not only provide structural support and promote healing in damaged areas but also enable the controlled release of loaded substances (such as cells, exosomes, and drugs) specifically at the injury site. Fan et al. (2020) prepared chitosan hydrogels loaded with exosomes to promote bone repair, demonstrating excellent osteogenic properties (Fan et al., 2020).

## 2 Exosome

### 2.1 Definition and identification methods

Exosomes are double-membrane vesicular nanoparticles in the category of extracellular vesicles, ranging in size from 30 to 150 nm, and are released from cells through a specific multi-step exocytosis process (Kalluri and LeBleu, 2020). Various cells and body fluids in the human body can secrete exosomes, including endothelial cells, immune cells, platelets, and smooth muscle cells. When secreted from donor cells to recipient cells, exosomes can regulate the biological activity of recipient cells through the proteins, nucleic acids, and lipids they carry. Due to their stability, biocompatibility, and multifunctionality, exosomes have garnered significant attention for their applications in regenerative therapy and tissue repair (Khazaei et al., 2023). Methods to identify exosomes in the human body typically require three techniques: transmission electron microscopy, particle size analysis, and surface marker detection (Welsh et al., 2024). The mainstream extraction method is ultracentrifugation, but other methods include ultrafiltration, high-performance liquid chromatography, polymer precipitation, affinity chromatography, and microfluidic systems.

### 2.2 Function

#### 2.2.1 Stem cell exosomes

##### 2.2.1.1 Exosomes derived from adipose-derived stem cells (ADSCs)

Adipose-derived stem cells (ADSCs) are mesenchymal stem cells derived from adipose tissue, possessing self-renewal capabilities and the ability to differentiate into osteoblasts, chondrocytes, and adipocytes. Exosomes derived from ADSCs also exhibit the ability to rapidly promote tissue repair while showing lower immunogenicity. ADSC-derived exosomes contain various bioactive substances, including growth factors such as transforming growth factor-beta (TGF-β), hepatocyte growth factor (HGF), and vascular endothelial growth factor (VEGF), all of which promote angiogenesis and tissue repair. ADSC-derived exosomes also contain anti-inflammatory cytokines that help regulate immune responses and inflammation, as well as matrix metalloproteinases involved in tissue remodeling (Papadopoulos et al., 2024). In wound healing, exosomes derived from ADSCs primarily function by regulating immune responses and inflammation, promoting angiogenesis, accelerating skin cell proliferation and re-epithelialization, and modulating collagen remodeling to inhibit scar formation. ADSC-derived exosomes also contain nucleic acids, such as microRNAs (miRNAs) and long non-coding RNAs (lncRNAs), which control post-transcriptional gene expression, as well as messenger RNA (mRNAs) that can be translated into functional proteins, inducing various cellular functions in recipient cells (Papadopoulos et al., 2024). In the fields of dermatology and plastic surgery, exosomes derived from human adipose stem cells are rich in miRNA-125a and miRNA-31, which can be transferred to vascular endothelial cells to stimulate proliferation and promote angiogenesis (Liang et al., 2016). ADSC-derived exosome miR-192-5p targets the receptor for the proinflammatory cytokine interleukin-17A (IL-17RA), regulating the Smad pathway in proliferative scar fibrosis, demonstrating great potential for the treatment of clinical proliferative scars (Li et al., 2021). In bone repair applications, a study has demonstrated that ADSC-derived exosomes effectively regulate bone immune metabolism through the immune modulation of miR-451a, further promoting bone healing and providing a therapeutic direction for bone repair (Li et al., 2022a). Currently, ADSC-derived exosomes have shown positive results in preclinical studies, demonstrating good tolerance and safety (Liu et al., 2023; Hu et al., 2022). However, their clinical application still faces challenges related to specificity, isolation methods, safety protocols, and the establishment of pharmacokinetic and pharmacodynamic properties (Papadopoulos et al., 2024).

##### 2.2.1.2 Exosomes derived from bone marrow mesenchymal stem (BMSC) cells

Bone marrow mesenchymal stem cells (BMSCs) are multipotent stem cells with self-renewal, immune modulation, and multilineage differentiation potential. They are easily accessible, have low immunogenicity, and can secrete nutritional factors, thus playing a significant role in bone tissue repair and showing immense potential for treating bone metabolic diseases (Kuang et al., 2019; Lu et al., 2020). Exosomes, as key mediators of intracellular signaling during the paracrine process of BMSCs, which involves the secretion of chemical factors that promote tissue repair and regeneration, exhibit similar therapeutic effects and functional characteristics while lacking the drawbacks of immune rejection (Chang et al., 2021; Riazifar et al., 2017). BMSC-derived exosomes (BMSC-Exos) contain various anti-inflammatory factors and growth factors, such as TNF-stimulated gene 6 (TSG-6), transforming growth factor-beta (TGF-β), interleukin-10 (IL-10), and tumor necrosis factor-alpha (TNF-α) (Wang et al., 2021a). BMSC-Exos can effectively promote cartilage repair and extracellular matrix synthesis (He et al., 2020). miRNAs, as one of the important cargoes carried by BMSC-Exos, have been widely studied for their potential to promote or inhibit bone repair. Research has shown that BMSC-Exos promote the polarization of M1 macrophages to M2 macrophages via miR-23a-3p, reducing early inflammatory responses at the tendon-bone interface and facilitating early healing after anterior cruciate ligament reconstruction surgery (Li et al., 2022b). Another study has shown that multiple miRNAs enriched in BMSC-derived extracellular vesicles may regulate osteogenic differentiation through the Wnt/β-catenin and BMP/Smad signaling pathways. Additionally, BMSC-EVs activate the Ras/Erk pathway, leading to significant angiogenesis, proliferation, and migration, thereby promoting fracture healing (Zhou et al., 2022). In the repair of spinal cord injuries, BMSC-derived exosomes offer new approaches to tissue repair. The miR-219-5p contained in BMSC-derived exosomes alleviates neuronal ferroptosis through the UBE2Z/NRF2 pathway, aiding in spinal cord injury recovery. Exosomes derived from human bone marrow mesenchymal stem cells reduce damage to the blood-spinal cord barrier after acute spinal cord injury via the TIMP2/MMP pathway. However, due to the limited osteogenic induction capacity of exosomes, their standalone use has shown poor results in bone regeneration (Pegtel et al., 2010). Modifying the contents of BMSC-derived exosomes through cell engineering techniques to create engineered exosomes, such as genetic modification or protein modification, or loading exosomes onto scaffolds, may help overcome the application limitations of BMSC-derived exosomes (Zhou et al., 2022).

##### 2.2.1.3 Exosomes derived from human umbilical cord mesenchymal stem cells (hUC-MSCs)

HUC-MSCs are a type of multipotent stem cell found in neonatal cord tissue. They possess strong proliferation and self-renewal abilities and can differentiate into one or more types of human tissues or organs under certain conditons. These cells offer advantages such as rapid self-renewal, low immunogenicity, and no ethical issues for research testing and medical usage (Zhang et al., 2022). Compared to other MSCs, hUC-MSCs exhibit stronger anti-inflammatory effects and enable more effective cartilage regeneration (Lee et al., 2021). Exosomes derived from human umbilical cord stem cells (hUC-MSCs) exhibit biochemical functions similar to hUC-MSCs and play a role in tissue repair by modulating inflammatory responses. A study has found that hUC-MSC-derived exosomes can reverse IL-1β-induced chondrocyte damage *in vitro* and regulate macrophage polarization (Li et al., 2022b). Additionally, another study has found that exosomes derived from human umbilical cord mesenchymal stem cells can promote cartilage repair in rats, as indicated by an increase in type II collagen expression (Yang et al., 2024a). In spinal cord repair, a study has shown that exosomes activate Wnt/β-catenin signaling in the spinal cord, promoting spinal repair through anti-apoptotic and anti-inflammatory effects (Kang and Guo, 2022). Exosomes transfer miR-199a-3p/145-5p to neurons in rats with spinal cord injury, affecting the ubiquitination of the high-affinity nerve growth factor receptor, tropomyosin receptor kinase A (TrkA), and promoting the NGF/TrkA signaling pathway. This mechanistic action suggests that hUC-MSC-derived exosomes may be a promising therapeutic strategy for spinal cord injury (Wang et al., 2021b).

#### 2.2.2 Other cell exosome classses

##### 2.2.2.1 Macrophage-derived exosomes

Macrophages exist in two different subpopulations: M1 and M2 macrophages. The balance between M1/M2 macrophage polarization determines the fate of organs and tissues during inflammation or injury. When infection or inflammation is severe enough to affect an organ, macrophages initially adopt the M1 phenotype to release TNF-α, IL-1β, IL-12, and IL-23 to counteract the stimulus. However, sustained or excessive M1 macrophage activity—characterized by prolonged inflammatory responses—may lead to tissue damage. In contrast, M2 macrophages secrete large amounts of IL-10 and TGF-β to suppress inflammation, aiding in tissue repair, remodeling, angiogenesis, and maintaining homeostasis. M1 macrophage-derived exosomes can promote inflammation, providing direction for anti-tumor strategies in treatment intervention (Gunassekaran et al., 2021). M2 macrophage-derived exosomes contain various cytokines and growth factors that promote wound healing and can actively target inflammatory sites while modulating the immune microenvironment. Studies indicate that exosome-induced macrophage phenotypic conversion exhibits excellent cell reprogramming ability and innate biocompatibility (Zhang et al., 2024; Kim et al.). By regulating the balance between pro-inflammatory and anti-inflammatory macrophages, a study presented a promising therapeutic approach for various inflammation-related diseases (Kim et al., 2019). M2 macrophage-derived exosomes also play a significant role in wound healing. Research has found that miR-21-5p is transferred to human umbilical vein endothelial cells (HUVECs) via M2 exosomes, acting as a key mediator to inhibit the tumor suppressor gene phosphatase and tension homolog (PTEN) and activate the AKT/mTOR signaling pathway, thereby regulating endothelial cell function. Injecting M2 exosomes into mouse full-thickness skin wounds can enhance angiogenesis and accelerate skin healing (Lyu et al., 2022). Additionally, M2 exosomes activate Wnt/β-catenin signaling through the transfer of the protein OTU deubiquitinase with linear linkage specificity (OTULIN), positively regulating angiogenesis and neurological recovery after spinal cord injury, demonstrating significant potential in spinal cord repair. Research has shown that M2 exosomes effectively attenuate bone necrosis, suppress the expression of pro-inflammatory mediators, promote osteogenesis and angiogenesis, reduce osteoclast formation, and modulate M1/M2 macrophage polarization in steroid-induced femoral head necrosis, making them one of the strategies for preventing steroid-induced femoral head necrosis (Yuan et al., 2024).

Cell-derived exosomes possess physiological functions similar to their parent cells and contain various bioactive factors that are crucial for cellular signaling, immune responses, angiogenesis, tissue healing, and other biological processes. They do not exhibit the drawbacks of stem cell therapies, such as reduced cell viability and regenerative capacity, immune rejection, and ethical concerns. In recent years, exosomes from various sources have been widely used for tissue repair and regeneration, gaining significant attention in the clinical application for various diseases, treatments, and diagnostics. However, the local efficacy of exosomes *in vivo* is low; therefore, combining exosomes with scaffolds for sustained release can expand their application in tissue repair.

#### 2.2.3 Plant exosomes

Plant exosomes are extracellular vesicles formed and released by plant cell membranes, carrying various biomolecules such as small RNAs, proteins, lipids, and secondary metabolites (Dad et al., 2021). These components enable plant exosomes to play a key role in tissue repair. Small RNAs can target and regulate gene expression in damaged cells, while proteins and lipids can promote repair by maintaining cell membrane stability and delivering growth factors (Mu et al., 2023). Additionally, secondary metabolites in plant exosomes possess antioxidant and anti-inflammatory properties, thus alleviating oxidative stress and inflammatory responses in tissues. By regulating gene expression, releasing bioactive molecules, and reducing oxidative stress, plant exosomes offer new strategies and tools for tissue repair. Research by Teng et al. (2021) demonstrated that exosomes derived from ginger, containing the ginger-derived miRNA aly-miR396a-5p, significantly reduce lung inflammation. The mechanism of action involves exosome-mediated delivery of aly-miR396a-5p and rlc-miR-rL1-28-3p, which prevent excessive inflammation by inhibiting the expression of Nsp12 and spike genes, respectively (Teng et al., 2021). Similarly, Xu et al. (2021) found that exosomes extracted from ginseng roots can stimulate the neural differentiation of bone marrow mesenchymal stem cells through the PI3K signaling pathway. This discovery suggests that plant exosomes may have significant potential in regenerative medicine for the nervous system (Xu et al., 2021). Furthermore, Hajialyani et al. (2018) found that exosomes extracted from wheat promote wound healing *in vitro* by enhancing cell viability and migration efficiency, as well as facilitating the formation of new blood vessels (Hajialyani et al., 2018). Despite the potential of plant exosomes in tissue repair, research on their mechanisms in plants remains limited compared to animal cell exosomes. This is largely due to the Complex composition and function of plant biology and the lack of breakthroughs in related technologies (Sarasati et al., 2023).

Research on both plant exosomes and animal cell exosomes in the field of tissue repair demonstrates their potential in promoting healing and regeneration. However, their compositions and mechanisms of action differ significantly. Plant exosomes primarily contain unique biomolecules, such as specific types of small RNAs, plant proteins, lipids, and secondary metabolites (e.g., flavonoids and phenolic acids). These components possess distinct biological functions and repair mechanisms. In contrast, animal cell exosomes consist of various small RNAs, membrane proteins, lipids, and extracellular matrix components, are more effective than plant-derived mechanisms in regulating intercellular signaling and tissue repair in human wound healing. These differences reflect the physiological differences and mechanisms faced by plants and animals in the process of tissue repair. Therefore, the application of plant exosomes and animal exosomes in the field of treatment and repair also needs to be adjusted accordingly according to their unique biological characteristics.

## 3 Hydrogels

### 3.1 Definition

Hydrogels consist of a three-dimensional space network that can absorb a significant amount of water due to the presence of hydrophilic groups (e.g., -NH). This allows them to swell in water while maintaining their structural integrity (Caló and Khutoryanskiy, 2015). The network of hydrogels is typically composed of cross-linked polymer chains or formed through cross-linked colloidal clusters, allowing water and small molecules to flow freely within. These polymer chains can absorb and retain large amounts of water, resulting in low hardness when dry, and a soft gel-like state when hydrated. The properties and behavior of hydrogels are influenced by various factors, including the type of polymer, cross-linking density, and degree of hydration. Due to their high water content, excellent biocompatibility, and controllable porosity, hydrogels have garnered significant attention in various fields, such as Medical & Biomedical Materials, Food Industry, Environmental Protection & Water Treatment and Cosmetics & Personal Care (Ahmed et al., 2013). Currently, hydrogels are primarily applied in drug delivery, tissue engineering, wound dressings, cosmetic products, as well as in sensors and smart materials.

### 3.2 Classification

Hydrogels can be classified into physical hydrogels and chemical hydrogels based on the type of bonding (Lu et al., 2018). Physical hydrogels are formed through physical forces such as electrostatic interactions, hydrogen bonding, and chain entanglement. They can exhibit reversibility, such as self-healing and responsiveness to environmental changes. Environment-sensitive hydrogels can detect slight variations or stimuli in external conditions (e.g., temperature, pH, light, pressure) and undergo corresponding changes in their physical structure and chemical properties (Luo et al., 2024; Soppimath et al., 2002). Shang et al. (2023) designed a thermoresponsive hydrogel by stabilizing N-vinyl caprolactam in water through carboxymethyl cellulose emulsification, followed by free radical polymerization with acrylamide (AM) under the initiation of ammonium persulfate (APS) to create CMC/poly (NVCL-co-AM) hydrogels. Urea was loaded as a model fertilizer, and its release behavior was studied at 25°C and 37°C to demonstrate the potential application of temperature-responsive hydrogels in controlled release of water and fertilizers. The results showed that as the temperature increased, the hydrogel swelled and released urea and water, providing ample supply of moisture and nutrients to plants. Due to these unique properties, hydrogels can closely mimic living tissues (Shang et al., 2023). Common physical naturally-occurring hydrogels include alginate and polyvinyl alcohol (PVA) hydrogels. Chemical hydrogels, on the other hand, are 3D network polymers formed through chemical cross-linking and are permanent in nature after development. They can be further classified into cross-linked and copolymer hydrogels. Cross-linked hydrogels connect polymer chains using chemical cross-linking agents, such as polyacrylamide hydrogels, which form a cross-linked network through agents like methylene bisacrylamide (MBA), providing strong mechanical strength and biocompatibility. Copolymer hydrogels are formed by copolymerization reactions that link different monomers into a cross-linked network, such as hydrogels created by the co-polymerization of polymers and cross-linking agents polyvinyl alcohol hydrogels, created by co-polymerizing PVA with cross-linkers like glutaraldehyde, exhibiting excellent mechanical properties and biocompatibility. While chemical hydrogels have a more stable polymer structure, the cross-linking agents used may be toxic. In contrast, physical hydrogels provide an alternative solution to the toxicity associated with chemical cross-linkers (Lu et al., 2018).

### 3.3 Loading and sustained release

The unique physical and chemical properties of hydrogels make them ideal carriers for loading and sustained release of exosomes. The loading and release of exosomes beneficially utilize the high water content and adjustable network structure of hydrogels (Annabi et al., 2014). The 3D network structure of hydrogels provides a supportive physical space to allow exosomes to be loaded into the hydrogel through physical adsorption, chemical crosslinking, or embedding (Riau et al., 2019). Once loaded, the exosomes can exist stably within the hydrogel and can be released through various mechanisms.

Exosome physical adsorption refers to the attachment of exosomes to the surface or within the hydrogel through non-covalent interactions, such as hydrogen bonds, electrostatic forces, and van der Waals forces (Thomas et al., 2008). This method of adsorption is physically simple for the exosomes, with mild loading conditions that do not cause chemical damage to the exosomes. However, the loading capacity is relatively high, so the release rate may be unstable and easily impacted by environmental conditions (such as pH and ionic strength). Exosome physical adsorption is suitable for applications that require rapid loading and release of exosomes, such as short-term drug delivery or emergency treatment. The chemical crosslinking method combines exosomes with the polymer chains of the hydrogel through covalent bonds. A common approach is to use crosslinking agents or chemical reactions to directly embed the exosomes within the hydrogel’s network structure. Chemical crosslinking loading provides higher stability and control of the loading, effectively extending the release time of the exosomes. However, it may also pose a risk of chemical damage to the exosomes, requiring precise control of reaction conditions. This method is suitable for applications that require long-term sustained release and stable loading, such as slow-release drug systems and long-term tissue engineering. Meanwhile, the embedding method involves directly incorporating exosomes during the formation of the hydrogel (Qin et al., 2016). This method typically occurs during the synthesis of the hydrogel, where exosomes are loaded within the gel network. It allows for a uniform distribution of exosomes and stable loading; the release behavior can be controlled by adjusting the crosslinking density and pore size of the hydrogel. However, with this method, it is essential to maintain the stability of the exosomes during the gelation process to prevent loss of activity during synthesis. This loading method can be utilized in tissue engineering and biomedical applications where stable and uniformly distributed exosomes are required. When choosing a specific method of loading exosomes, factors such as the stability of the exosomes, release requirements, ease of operation, and the demands of the final application should be considered. A well-designed loading strategy will enhance the efficacy and performance of the exosomes in their applications (Riau et al., 2019).

The sustained release process typically involves the gradual release of exosomes from the hydrogel network. The mechanisms of sustained release encompass various physicochemical processes, including diffusion, permeation, environmental responsiveness, chemical interactions, mechanical release, and chemical degradation (Lin and Metters, 2006). The sustained release process not only depends on the interactions between exosomes and hydrogel, but also on the properties of the hydrogel itself. According to Fick’s diffusion law, hydrogels with larger pore sizes allow for faster diffusion of exosomes, while smaller pore sizes restrict their release rate (Peppas et al., 2000). Exosomes’ release from hydrogels typically begins with their dissolution or dispersion within the hydrogel, followed by diffusion through the gel’s pores into the external environment. The diffusion rate is influenced by the pore size of the hydrogel, the size of the exosomes, and the hydration properties of the hydrogel (Nicol, 2021). The permeation mechanism refers to the process where water molecules enter the hydrogel, causing it to swell and thereby promoting the release of exosomes. The environmental response mechanism involves the hydrogel reacting to external stimuli (such as pH, temperature, or ionic strength), which triggers the release of exosomes. For example, pH-responsive hydrogels swell in acidic environments and contract in alkaline conditions, affecting the release rate of exosomes. Temperature-responsive hydrogels expand at high temperatures or contract at low temperatures, thereby controlling the release of exosomes (Soppimath et al., 2002). The chemical interaction mechanism refers to the interactions between exosomes and the chemical groups or crosslinkers in the hydrogel that influence their release process. For example, charged exosomes may bind to oppositely charged hydrogel groups through electrostatic adsorption, potentially fixing the exosomes within the hydrogel and affecting their release. The mechanical release mechanism involves the mechanical changes in the hydrogel that impact exosome release. Lastly, the chemical degradation mechanism pertains to how the hydrogel’s chemical breakdown affects the release of exosomes (Vlugt-Wensink et al., 2006). By understanding and optimizing these mechanisms for specific scenarios, it is possible to design hydrogel systems with well-controlled release properties that meet the needs of various biomedical applications.

## 4 Applications of different exosomes loaded in hydrogels

Hydrogels, with mechanical and structural properties similar to many tissues and extracellular matrices (ECM), have garnered significant attention in the field of tissue repair Substantial advancements have been made in the design, synthesis, and application of these materials for various biological and biomedical uses. Many surface molecules on exosomes enable their internalization through receptor-mediated endocytosis, playing a crucial role in regulating intercellular communication (Pegtel and Gould, 2019). However, the stability and retention of exosomes released *in vivo* pose significant challenges, as they are rapidly cleared by the innate immune system (Riau et al., 2019). To overcome the rapid clearance of exosomes and maintain their bioactivity, hydrogels have been utilized to encapsulate these small vesicles, providing protection and enabling controlled release.

### 4.1 Combination of exosomes derived from ADSCs with hydrogels

The reliability of combining exosomes derived from ADSCs with hydrogels for tissue repair has been supported by numerous studies. ADSC-derived exosomes contain key factors related to angiogenesis and damaged tissue regeneration. Researchers have designed a hydrogel loaded with adipose stem cell exosomes that exhibits anti-infection and microenvironment protection properties, hence promoting regeneration of the thin endometrial layer. The resulting hydrogel is injectable and demonstrates antibacterial and self-healing characteristics consistent with enhanced endometrial regeneration. It is also suitable for minimally invasive injections into the uterine cavity, facilitating accelerated tissue healing. The self-healing properties of this hydrogel allow it to maintain a stable structure even in the presence of rhythmic endometrial contractions and shedding (Lin et al., 2021). Li et al. (2022a) conducted a study by loading ADSC-derived exosomes into a nano-hydrogel and implanting it into the calvaria of rats. The research demonstrated that exosome-loaded gelatine nanoparticles effectively modulate bone immune metabolism and promote bone healing through the immunoregulatory role of miR-451a (Li et al., 2022a). Chen et al. (2019) also embedded exosomes derived from miR-375-overexpressing ADSCs into hydrogels and implanted them into calvaria defect areas of the rats. The results indicated that miR-375-enriched ADSC-exosomes promote bone regeneration (Chen et al., 2019). Using gene modification to obtain exosomes enriched with specific miRNAs can enhance their function and combine them with hydrogels for controlled release can better achieve the goal of tissue repair.

### 4.2 BMSC-derived exosomes combined with hydrogels

In recent years, BMSC-derived exosomes have emerged as a novel acellular therapeutic platform for various diseases due to their therapeutic effects, including promoting regeneration and modulating immune responses, while carrying multiple therapeutic growth factors and miRNAs. BMSC-derived exosomes can promote axonal regeneration and angiogenesis, improve structural and electrophysiological outcomes, and reduce neuroinflammation and glial proliferation, significantly enhancing motor and sensory recovery following spinal cord injury (Guo et al., 2019; Li et al., 2020). Fan et al. (2022) designed a conductive hydrogel loaded with bone marrow mesenchymal stem cells, which was injected into the spinal cord injury sites of mice. The results showed that delivering BMSC-derived exosomes within the conductive hydrogel could mitigate adverse host immune responses while synergistically promoting neuronal and axonal regeneration, thereby alleviating spinal cord injuries (Fan et al., 2022). Due to its tissue-like softness and the presence of an inherent electric field similar to that of the innate nervous system, conductive hydrogels can provide mechanical and electrical signals to enhance the neuronal differentiation of neural stem cells and control neurite extension. Conductive hydrogels mimic the electrical transmission properties of natural nervous tissue, making them highly beneficial for spinal cord injury repair. Therefore, the combination of conductive hydrogels with bone marrow mesenchymal stem cells holds great potential for applications in spinal cord repair (Fan et al., 2022; Wu et al., 2016; Tseng et al., 2015). BMSC-derived exosomes enrich genes which upregulate the osteogenic differentiation of BMSCs. He et al. (2023) developed a temperature-sensitive hydrogel loaded with BMSC exosomes, which was injected into a rat bone defect model. The results also indicated that BMSC exosomes can promote bone regeneration, highlighting their promise as a biomaterial for the regeneration of new bone (He et al., 2023).

### 4.3 Combination of hUC-MSC -derived exosomes with hydrogels

Exosomes derived from hUC-MSCs promote angiogenesis, thereby accelerating skin wound healing (Zhang et al., 2015). Yang et al. (2020) designed a temperature-sensitive hydrogel loaded with hUC-MSC-derived exosomes. At low temperatures, the gel is in a liquid state, while at high temperatures, it becomes a solidified gel, adapting to the complex and irregular spaces of diabetic foot wounds. This allows bioactive agents to adhere to the target site and exert their biological effects. Experimental results showed that the hydrogel loaded hU-CMSC-derivedd exosomes significantly accelerated wound closure rates, enhanced granulation tissue regeneration, and upregulated the expression of vascular endothelial growth factor (VEGF) and transforming growth factor β-1 (TGFβ-1) (Yang et al., 2020). Some studies have also loaded exosomes into hydrogels, followed by freeze-drying for transportation. The hydrogels can then be dissolved to create dressings. For example, Results of a study indicate that these gels can promote scar-free wound healing (Yang et al., 2024b). Hu et al. (2021) designed a hydrogel loaded with hUC-MSC exosomes, which was injected into a mouse cranial defect model. The study found that hUC-MSCs-EVs delivered miR-23a-3p, activating the PTEN/AKT signaling pathway to promote osteogenesis and angiogenesis, achieving vascularized bone regeneration (Hu et al., 2021).

### 4.4 M2 macrophage-derived exosomes combined with hydrogel

The exosomes derived from M2 macrophages, with overexpression of miR-223 (ExosM2@miR-223), are believed to play a significant role in angiogenesis and promote wound healing. Yuan et al. (2024) designed an injectable, self-healing, and adhesive hydrogel aimed at accelerating diabetic wound repair. By eradicating bacterial infections, alleviating oxidative stress, and providing a sustained release of MnO2 and Exos, the hydrogel loaded with MnO2 and M2 macrophage-derived exosomes created a suitable microenvironment to inhibit inflammation, promote cell proliferation, restore angiogenesis, and enhance granulation tissue formation and epithelial healing, overall showing significant improvement in the healing of diabetic wounds *in vivo* (Xiong et al., 2021). Additionally, Kwak et al. (2022) loaded M2 macrophage-derived exosomes into Polyethylene glycol hydrogels and implanted them in animal models. The experimental results demonstrated that the localization of M2-Exos facilitated a successful transition from M1 macrophages to M2 macrophages within the lesions for over 6 days (Kwak et al., 2022). This approach enhanced therapeutic effects in animal models, including accelerated wound closure and improved healing quality, ultimately promoting skin wound healing. M2 macrophage-derived exosomes also promote osteogenic differentiation. Yu et al. (2022) designed a HA@SDF-1α/M2D-Exos hydrogel that enables the synchronized, sustained release of SDF-1α and M2D-Exos, enhancing the proliferation and migration of HMSCs and human umbilical vein endothelial cells (HUVECs). This approach promotes osteogenesis and angiogenesis both *in vitro* and *in vivo*, providing a novel strategy for accelerating bone repair (Shang et al., 2023; Yu et al., 2022).

### 4.5 Plant-derived exosomes combined with hydrogel

Plant exosomes are nanoscale vesicles released by plant cells, containing biomolecules such as proteins, lipids, and RNA, which can regulate intercellular communication. Loading plant exosomes into hydrogels involves embedding these exosomes within the hydrogel network structure for tissue repair applications. Plant exosomes contain growth factors and cell signaling molecules that can promote cell differentiation and assist in the regeneration and repair of damaged tissues. They also possess anti-inflammatory properties, regulating immune responses and alleviating inflammation, further supporting the tissue repair process. Additionally, plant exosomes enhance cell migration, improving the migration of cells in damaged areas for more effective tissue repair. Xu et al. (2021) designed a photosensitive hydrogel loaded with ginseng exosomes, showing strong efficacy in guiding the neural differentiation of BMSCs (Xu et al., 2021).

Due to challenges in the extraction and purification techniques of plant exosomes, as well as factors affecting their stability, research on plant exosomes lags behind that of animal exosomes. Research on loading plant exosomes into hydrogels is still in the developmental stage, but its prospects are promising. With advancements in material science and biotechnology, more innovations may emerge in this field. For example, utilizing the properties of smart hydrogels could enable the design of drug release systems that respond to external stimuli, enhancing treatment personalization and targeting. Additionally, the potential applications of plant exosomes could extend to food preservation and the development of functional foods, providing new ideas for modern agriculture and the food industry.

## 5 Conclusion

Exosomes exhibit numerous advantages in the field of tissue repair, primarily in promoting cell regeneration and regulating immune responses. They can carry bioactive molecules such as growth factors, miRNA, and proteins, which stimulate cell proliferation and differentiation, facilitating the repair of damaged tissues. For instance, exosomes derived from stem cells have been shown to promote the regeneration of cardiac, neural, and bone tissues. Additionally, exosomes play a crucial role in regulating inflammatory responses by transmitting anti-inflammatory signals, thereby reducing local inflammation and creating a more favorable microenvironment for repair. This characteristic gives exosomes significant potential in treating chronic inflammatory diseases and aiding recovery after injuries. The application range of exosomes in promoting human wound recovery is extensive.,including enhancing tissue regeneration, reducing inflammation, and accelerating healing in skin injuries and chronic wounds. In bone tissue repair, exosomes can promote the proliferation and differentiation of osteoblasts, enhancing the speed of bone healing. In the repair of nerve injuries, exosomes assist in nerve regeneration and functional recovery. Furthermore, exosomes can serve as biomaterials that integrate with implants, improving tissue integration.

The combination of exosomes and hydrogels offers multiple advantages, drawing significant attention in the research fields of tissue repair and regenerative medicine. First, hydrogels, such as biomaterials, possess excellent biocompatibility and biodegradability, providing an ideal growth environment for cells. Their 3D network structure effectively supports cell adhesion and proliferation. Secondly, the combination of hydrogels and exosome allows for sustained release, as hydrogels can slowly release encapsulated exosomes, prolonging their biological activity and continuously providing growth factors and signaling molecules to promote tissue repair. Additionally, the bioactive components in exosomes can enhance the properties of hydrogels, such as increasing their mechanical strength and bioactivity, making them more suitable for tissue engineering. Furthermore, exosomes can regulate cell behavior within hydrogels, promoting cell proliferation, migration, and differentiation, significantly improving tissue regeneration outcomes. Lastly, the combination enables targeted therapy by adjusting the composition and structure of hydrogels, improving drug delivery systems, and enhancing treatment precision and efficacy. In summary, the multiple advantages of combining exosomes with hydrogels provide new research directions and application possibilities for tissue repair and regenerative medicine.

While exosomes show potential in tissue repair applications, they also face several limitations. First, the purification and quantification of exosomes remain challenging, affecting their consistency and reproducibility in clinical use. Additionally, variations in the source and characteristics of exosomes may lead to uncertain efficacy, as exosomes from different cell types exhibit differences in bioactivity. When combined with hydrogels for tissue repair, issues such as uneven release rates, difficulties in quantitative assessment, and decreased exosome stability can limit clinical application. Future efforts to optimize exosome isolation and purification techniques, along with standardizing characterization and functional assessments, can expand their application scenarios. Furthermore, optimizing hydrogel composition and using materials with varying porosity and crosslinking can help control the release rate of exosomes. Improving exosome preservation and handling methods, such as employing low-temperature storage or encapsulation techniques, can enhance their stability. Ultimately, these improvements could significantly enhance the effectiveness of exosome-hydrogel combinations in tissue repair, broadening the application prospects of exosomes in the field of tissue repair.
